# Pretransplant Evaluation and Liver Transplantation Outcome in PBC Patients

**DOI:** 10.1155/2022/7831165

**Published:** 2022-07-22

**Authors:** Maja Mijic, Ivona Saric, Bozena Delija, Milos Lalovac, Nikola Sobocan, Eva Radetic, Dora Martincevic, Tajana Filipec Kanizaj

**Affiliations:** ^1^Department of Gastroenterology, University Hospital Merkur, Zajceva 19, Zagreb, Croatia; ^2^University of Zagreb, School of Medicine, Salata 3, Zagreb, Croatia

## Abstract

Primary biliary cholangitis (PBC) is an autoimmune chronic cholestatic liver disease characterized by progressive cholangiocyte and bile duct destruction leading to fibrosis and finally to liver cirrhosis. The presence of disease-specific serological antimitochondrial antibody (AMA) together with elevated alkaline phosphatase (ALP) as a biomarker of cholestasis is sufficient for diagnosis. Ursodeoxycholic acid (UDCA) is the first treatment option for PBC. Up to 40% of patients have an incomplete response to therapy, and over time disease progresses to liver cirrhosis. Several risk scores are proposed for better evaluation of patients before and during treatment to stratify patients at increased risk of disease progression. GLOBE score and UK PBC risk score are used for the evaluation of UDCA treatment and Mayo risk score for transplant-free survival. Liver transplantation (LT) is the only treatment option for end-stage liver disease. More than 10 years after LT, 40% of patients experience recurrence of the disease. A liver biopsy is required to establish rPBC (recurrent primary biliary cholangitis). The only treatment option for rPBC is UDCA, and data show biochemical and clinical improvement, plus potential beneficial effects for use after transplantation for the prevention of rPBC development. Additional studies are required to assess the full impact of rPBC on graft and recipient survival and for treatment options for rPBC.

## 1. Introduction

Primary biliary cholangitis (PBC) is an autoimmune, chronic, cholestatic liver disease characterized by progressive cholangiocyte destruction, eventually leading to intrahepatic bile duct destruction, fibrosis, and liver cirrhosis [1]. The pathogenesis of the disease is not completely understood but is caused by an interplay of environmental, immunogenetic, and epigenetic factors [1]. In the last decade, published studies gave better insight regarding PBC prevalence and incidence even though the results varied largely depending on the region, local awareness, and diagnostic possibilities. According to available data, the estimated prevalence ranges from 1.9 to 39.2 and incidence from 0.3 to 5.8 per 100,000 population per year in Europe, and the estimated prevalence and incidence for North America range from 2.24 to 40.2 and from 0.33 to 3.03 per 100,000 population per year with reported female-to-male ratio as high as 10 : 1, respectively [2]. It is usually diagnosed in the 5^th^ or 6^th^ decades of life [2]. Increasing prevalence and incidence are mostly due to easier diagnosis of the disease since the discovery of disease-specific serological antimitochondrial antibodies (AMAs). The presence of AMA together with elevated alkaline phosphatase (ALP) as a biomarker of cholestasis is sufficient for diagnosis [3]. AMA is detected in approximately 95% of PBC patients, rarely in other diseases, and analysis is available worldwide [1, 3]. Liver biopsy is necessary only in the absence of AMA in cases with a high suspicion of PBC or other chronic parenchymal liver diseases [1]. The natural history of the disease is progressive but unpredictable. Some patients rapidly progress to end-stage liver disease (ESLD), while others remain asymptomatic for decades. Early diagnosis and initiation of therapy can significantly improve the course of the disease. Ursodeoxycholic acid (UDCA), a natural hydrophilic bile acid, is the first treatment option for PBC, approved for use in 1997 [4]. UDCA improved survival rates for PBC patients and overall prognosis [1, 3, 4]. However, up to 40% of patients have an incomplete response to therapy and over time disease progresses to liver cirrhosis [5, 6]. Liver transplantation (LT) remains the only definitive treatment option for end-stage liver disease and its complications. Before the introduction of UDCA as a standard treatment for PBC patients, LT was the only treatment option for PBC and this chronic liver disease was the most common indication for LT in the 1980s [6]. Nowadays, LT has been used for the treatment of PBC-related cirrhosis and malignancy, a disease refractory to control by medication, or when symptomatic treatments fail to control pruritus [7]. Thus, several scoring systems have been presented to determine clinical outcomes and to stratify patients with increased risk of treatment failure and disease progression to liver cirrhosis. Symptomatic PBC patients have a median survival time of up to 10 years without LT, and once the decompensated disease develops, the median survival time decreases to 3 to 5 years [8].

## 2. Treatment

During the last two decades, the clinical course of PBC has significantly improved due to earlier disease recognition and widespread use of UDCA [3, 6, 7, 9–14]. Also, more frequent routine tests and improved AMA isolation methods led to the detection of clinically asymptomatic patients with normal liver enzymes. In 2017, European Association for the Study of the Liver (EASL) guidelines stated that AMA reactivity alone is not sufficient to diagnose PBC and recommend follow-up of these patients with annual biochemical reassessment for the presence of liver disease and in case of the biochemical activity or signs of chronic liver disease treatment should be initiated [1]. Many studies have reported that PBC patients who had early liver disease and were not treated with UDCA have a shortened survival in comparison with the healthy population regardless of symptoms [11, 12]. In three contemporary series, asymptomatic patients had a 10-year survival ranging from 50% to 70%. Additionally, symptomatic patients had a median duration of survival from 5 to 8 years from the onset of symptoms [11, 12]. Several trials have reported that UDCA is associated with significant improvement in liver function tests, improvement in histology, and prolonged transplant-free survival [3, 6, 9, 10]. For example, a French randomized trial that was published 20 years ago reported that the risk of progression from stages I-II to stages III-IV was 7% ± 2% in UDCA, while in the placebo group it was 34% ± 9% [13]. In an early prospective study of 180 patients, the authors investigated the usefulness of UDCA therapy in the prevention of esophageal varices development [14]. Patients received UDCA vs. placebo and were monitored for up to 4 years. The authors reported that the risk of developing varices was 16% for the UDCA-treated patients, while it was 58% for those receiving the placebo [14]. UDCA is a synthetic bile acid that has anti-inflammatory properties, promotes bile excretion, and reduces the severity of cell injury [7].

Current guidelines recommend that the dose for PBC treatment is 13 to 15 mg/kg/d [6]. An ongoing clinical trial (NCT03345589) aims to investigate the efficacy of an intermediate dose of UDCA 18–22 mg/kg/day in comparison with the standard dose over 6 months of therapy. The trial endpoint is biochemical remission [7].

Although UDCA as a first-line treatment option for PBC treatment is associated with slowing the progression of chronic liver disease, this drug is ineffective for the common symptoms of fatigue or pruritus [6]. Moreover, up to 40% of PBC patients have partial or no response to UDCA. Failure to respond to UDCA is defined as a lack of normalization or reduction in ALP by greater than or equal to 40% at 1 year of UDCA treatment [7]. Risk factors that are associated with nonresponse to UDCA are age (females under 45 years), male gender, and the presence of advanced liver disease. Patients who have a poor response to UDCA will have a poor outcome [6].

Due to these limitations of UDCA, in 2016, a new drug named obeticholic acid (OCA) was introduced as a second-line treatment for PBC. OCA is a potent farnesoid X receptor (FXR) agonist [7]. OCA can be used as monotherapy in those PBC patients who do not tolerate UDCA or in combination with UDCA for those who are nonresponsive to UDCA [6, 7]. According to data, OCA is an effective adjunctive treatment for UDCA-refractory or UDCA-intolerant PBC. The current dosing guidelines for OCA were established by the POISE trial phase III, which analyzed 210 patients [15]. In this double-blind, placebo-controlled trial, PBC patients were receiving OCA 5 mg/day titrated to 10 mg after 6 months if lacking clinical benefit, or OCA 10 mg [15]. Patients were treated for one year. In this trial, the primary endpoint was an ALP level of less than 1.67 times the upper limit of the normal range, with a reduction of at least 15% from baseline, as well as normal bilirubin levels. The primary endpoint occurred in more PBC patients in the 5–10 mg group and the 10 mg group than in the placebo group (*p* < 0.001 for both groups). On the other hand, there was no significant difference in noninvasive fibrosis markers after 12 months of therapy between groups. Moreover, pruritus was more common with OCA than with placebo. There are still ongoing debates regarding the safety of OCA because there is some evidence that it can cause drug-induced liver injury (DILI), liver decompensation, or acute liver failure requiring LT [7]. So far, it looks like these side effects depend on the dose of the drug and the stage of liver disease [16]. In the ongoing phase 4 COBALT trial, the safety and efficacy of OCA are being investigated. The primary endpoints of this trial (NCT02308111) include death, transplant, and hepatic decompensation [7]. Although studies examining the efficacy of OCA on the survival of patients with PBC are still ongoing, based on the results, the recommended starting dose for patients with preserved synthetic function and in Child-Pugh class A cirrhotic patients is 5 mg daily. After 3 months, the dose can be increased to 10 mg daily if liver chemistries remain abnormal and the patient is tolerating the medication well [17]. On the other hand, Child-Pugh class B or C cirrhotic patients at the beginning of the trial were dosed at a max of 5 mg weekly. In May 2021, FDA (Food and Drug Administration) issued restrictions for the use of OCA in patients with decompensated cirrhosis, a prior decompensation event, or with compensated cirrhosis who have evidence of portal hypertension due to serious liver injury leading to liver decompensation or liver failure. More FXA agonists are under investigation for use in PBC (cilofexor (NCT02943447), tropifexor (NCT02516605), and EDP-305 (NCT03394924)) [18].

Another promising candidate as a second-line treatment for UDCA is fibrates, targeting peroxisome proliferator-activated receptors (PPARs), which affect bile acid synthesis and detoxification [7]. There are three isoforms of PPAR: *α*, *δ*, and *γ*. Several small-sized pilot studies showed an improved biochemical response (reduction in ALP levels to normal ranges and improved ALT levels) with the addition of 160 mg/day of fenofibrate (PPAR-*α*) to a standard dose UDCA [19]. In the retrospective study published by Cheung and colleagues, 41% of the patients in the fenofibrate and UDCA group met the criteria for clinical response (using Toronto criteria), versus 7% in the UDCA-only group [20]. Exposure to fenofibrate was associated with improved transplant-free and overall survival. On the other hand, more than 20% of patients stopped taking therapy due to side effects and there was a significant increase in bilirubin levels in patients with advanced fibrosis predisposing hepatic decompensation. Of all available studies on fibrates, the most important is the BEZURSO, a double-blind, randomized, placebo-controlled French study, including 100 patients with PBC and incomplete response to UDCA [21]. It demonstrated that a 2-year combination treatment of UDCA and bezafibrate (BZF) at 400 mg/day had a significantly higher rate of complete biochemical response defined by normal levels of ALP, total bilirubin, and aminotransferases (30% vs. none in the placebo group). Also, patients in UDCA and BZF groups had an improvement in liver fibrosis compared with placebo measured via vibration-controlled transient elastography, a decrease of 15% in liver stiffness measurement in the BZF group, compared with an increase of 22% in the placebo group, a difference of 36 percentage points (95% CI, 8–64) [21]. Improvement of symptoms including pruritus was also reported. Since PBC is a slowly progressive chronic disease, it is difficult to prove whether these beneficial effects on liver enzymes and symptoms of the disease can translate into lower overall liver-related mortality or the need for LT. In April 2021, a large retrospective Japanese study was published, in which the use of UDCA-BZF combination therapy, compared with UDCA only, was associated with a significant decrease in all-cause and liver-related mortality or need for LT (adjusted hazard ratios: 0.3253, 95% CI, 0.1936–0.5466 and 0.2748, 95% CI, 0.1336–0.5655, respectively; *p* < 0.001 for both) [22]. The number needed to treat with combination therapy to prevent 1 additional death or LT over 5, 10, and 15 years was 29 (95% CI, 22–46), 14 [10–22], and 8 [6–15], respectively. Fibrates are overall very well tolerated, with minor side effects of heartburn, myalgias, increase in serum creatinine, and transient transaminase elevations reported in clinical trials for PBC [4]. Recently, a dual PPAR-*α* and PPAR-*δ* agonist, elafibranor, is investigated as a second-line treatment for PBC patients with incomplete response to UDCA treatment [23]. In phase 2 placebo-controlled trial (NCT03124108), the addition of elafibranor for 12 weeks was found to significantly reduce ALP levels and improve lipid and anti-inflammatory markers [23]. Also, in phase II pruritus was not induced and patients with pruritus at the baseline reported less symptoms at the end of the treatment [23]. The results of phase III global trial are expected to assess the efficacy, safety, and tolerability of elafibranor relative to the currently approved second-line therapy for patients with PBC.

Even though PBC is an autoimmune-mediated liver disease, the addition of immunosuppressants (budesonide, mycophenolate mofetil, azathioprine, methotrexate) did not provide extra treatment benefits so far [18]. New treatment strategies targeting various stages of primary biliary cholangitis pathogenesis are investigated. However, these investigations are limited by the fact that PBC is a heterogeneous disease and hard endpoints take years to develop.

## 3. Risk Assessment

Risk assessment should evaluate disease severity and activity at baseline and during treatment using static and dynamic markers of the disease. Static markers important for disease prognosis are demographic characteristics (age at the time of diagnosis and sex), serological profiles (AMA or antinuclear antibodies (ANAs) present), laboratory markers of fibrosis (hyaluronic acid, enhanced liver fibrosis (ELF) score, the aspartate aminotransferase-to-platelet ratio index (APRI), noninvasive liver stiffness measurement (LSM), presence of portal hypertension, and histological features at the time of diagnosis. Younger age at the time of diagnosis (less than 45 years) is associated with more symptomatic patients who are less likely to respond to treatment and are at a higher risk of liver-related mortality [24], whereas the male sex is associated with higher age and more advanced disease at the time of diagnosis with a higher risk of hepatocellular carcinoma (HCC) development [25, 26]. Besides AMA, the autoantibody profile of PBC includes antinuclear antibodies (ANAs) also known as PBC-specific ANA (rim-like/membranous patterns (antibodies against gp210) and the multiple nuclear dots (sp100 antigens)) and their positivity strongly suggests the diagnosis of PBC, irrespective of AMA status [27, 28]. The presence of antibodies against gp210 and sp100 antigens is often associated with severe PBC and an unfavorable course of disease [29, 30], but their role as prognostic markers is yet to be determined. Several serum markers of fibrosis showed prognostic ability in PBCs such as hyaluronic acid, ELF score, and APRI index, but there are no data regarding the change in these parameters with time and their relationship with change in the disease characteristics [31]. The best noninvasive surrogate marker for the detection of cirrhosis and advanced fibrosis in patients with PBC is LSM assessed by transient elastography (TE). In 2012, Corpechot et al. showed that baseline values of LSM of 9.6 kPa and yearly LSM increase of 2.1 kPa are associated with a five- and eightfold increased risk of decompensation, liver transplantation, or death (95% CI: 1.5–15.9; *p* < 0.0001; 95% CI: 3.6–36.0; *p* < 0.0001) [32]. In 2021, EASL guidelines recommend the use of LSM to monitor PBC progression because it was shown that worsening of LSM has a higher predictive value for poor outcome in comparison with the LSM value at the baseline [33]. Another important marker of disease prognosis is the presence of portal hypertension and what we know so far is that portal hypertension can be present in the early stages of the disease long before cirrhosis development, but the underlying pathophysiological mechanism is poorly understood. In the research published by Warnes et al., 82% of the pre-cirrhotic PBC patients had portal hypertension (hepatic venous pressure gradient (HVPG) >5 mmHG) and 34% had HVPG >12 mmHg (clinically significant portal hypertension (CSPH)) [34]. To avoid unnecessary endoscopy screening for esophageal varices or invasive portal pressure gradient measurement, current Baveno VII guidelines recommend using TE and indirect signs of portal hypertension (thrombocytopenia, splenomegaly) to stratify patients who require upper endoscopy. Baveno VII guidelines use the term “compensated advanced chronic liver disease (cACLD)” based on LSM, and values between 10 and 15 kPa are suggestive of cACLD, and values >15 kPa are highly suggestive of cACLD [35]. Therefore, LSM by TE < 15 kPa plus platelet count >150 × 10^9^/L rules out CSPH (sensitivity and negative predictive value > 90%) in patients with cACLD. If LSM increases (>20 kPa) or platelet count declines (<150 × 10^9^/L), these patients should undergo screening endoscopy [35]. Current EASL guidelines on noninvasive markers suggest using a cutoff value of LSM ≤10 kPa to rule out cACLD in PBC patients [33]. Even though liver biopsy is no longer necessary for PBC diagnosis in the presence of AMA antibody and cholestatic liver biochemistry, it can still be useful in patients who have an inadequate response to UDCA or if there is a clinical suspicion of coexisting disease, especially autoimmune hepatitis. It is shown that certain histological findings are an independent predictor of cirrhosis development and poor response to UDCA treatment, such as the degree of lymphocytic interface hepatitis and the presence of ductopenia [36, 37]. Also, it is important to identify individuals with overlap syndrome because they could benefit from combined treatment with immunosuppressants and UDCA. Up to 10% of PBC patients may present with clinical features of other autoimmune liver diseases, especially autoimmune hepatitis (AIH), known as PBC AIH overlap syndrome [38]. Typical features of AIH can be present at the time of PBC diagnosis but sometimes can present sequentially even years after diagnosis of PBC. Two scoring systems have been used to evaluate patients with PBC AIH overlap syndrome. The first one, published by the International Autoimmune Hepatitis Group, was presented only for the diagnosis of AIH using four criteria (simplified version): autoantibodies, immunoglobulin G, histology, and exclusion of viral hepatitis, and additional studies showed that a score of 7 has overall sensitivity and specificity of 87.1% (95% CI: 84.5–87.6) and 99.6% (95% CI: 98.2–99.9) for AIH diagnosis and can be efficacious also for overlap syndrome [39]. The second one, the Paris criteria, is nowadays mostly used to identify overlap syndrome. According to these criteria, a diagnosis can be made in a patient with PBC with at least two of the following:

- Alanine aminotransferase activity >5 times the upper limit of normal
- IgG ≥2 times the upper limit of normal and/or positive anti-smooth muscle antibody
- Liver biopsy with moderate or severe interface hepatitis

These criteria were incorporated in the latest EASL guidelines for the management of patients with PBC. Both criteria require liver biopsy for the definitive diagnosis.

Since 1983, to estimate the prognosis of patients with PBC and response to UDCA treatment, several risk scores have been made, which could be generally divided into two groups: models that predict the survival of PBC patients in the pre-UDCA era and models of biochemical response predicting clinical outcomes in the UDCA era. Major PBC-specific prognostic models are summarized in Table 1.

### 3.1. Mayo Risk Score

With the absence of therapeutic intervention in 1983 and 1985, the Yale model and European model were the first PBC-specific prognostic scoring systems. Since none of these models could accurately calculate patient survival and both required liver biopsy, in 1989 Dickson et al. proposed the Mayo score (*R* = 0.039 × age in years + 0.871 × ln (bilirubin in mg/dL) + 0.859 × edema − 2.53 × ln (albumin in gm/dL) + 2.38 × ln (prothrombin time in seconds)) [42]. In the beginning, the model was less useful in predicting survival over time since it was based on baseline characteristics. In 1994, this model was revised and further simplified. The same variables were used (INR instead of PT) to predict short-term survival, described as less than 2 years of survival or time to transplantation at any time point during follow-up. In conclusion, scores greater than 7.8 were associated with a progressively increased post-LT mortality rate [51]. Nowadays, the model contains six variables: age, prothrombin time, bilirubin and albumin levels, presence or absence of edema, and dependence on diuretics. As it can be seen, the Mayo risk score has one great advantage—it does not require liver histology to calculate the risk score, which is among the many reasons why this score is still widely used.

### 3.2. UDCA Era

In the UDCA era, several groups have published different biochemical response criteria that predict overall survival and progression of liver disease based exclusively on treatment response, i.e., the Barcelona, Paris I, Rotterdam, Toronto, and Paris II criteria. Among all of them, only Toronto criteria were developed comparing histologic disease progression in the paired biopsies from the same patients with biochemical response to UDCA therapy. The Toronto criteria define biochemical response to UDCA as ALP less than 184 IU/L (1.67 × ULN) after 2 years of treatment. In paired liver biopsies, more than 80% of patients who did not respond to UDCA according to the criteria showed histologic progression after 10 years (odds ratio, 12.14; 95% CI, 2.69−54.74) [37]. The biochemical response criteria after 12 months of UDCA treatment are the most validated and easy to use. The Paris I criteria are generally considered the best to predict transplant-free survival for patients with advanced PBC (stages III-IV). Patients with ALP <3 ULN, AST <2 ULN, and bilirubin ≤17 *μ*mol/L after 1 year of UDCA had a 10-year transplant-free survival rate of 90% compared with 51% [44]. To predict the prognosis of patients with early-stage PBC, Paris II criteria were defined as AST and ALP ≤1.5 ULN, with a normal bilirubin level after 1 year of UDCA therapy [46]. French studies showed that among 165 early-stage PBC patients survival rates without adverse outcomes at 5, 10, and 15 years of follow-up were 100% in responders and 93%, 87%, and 74%, respectively, in nonresponders [46].

### 3.3. GLOBE Score

GLOBE score system was a model made in 2015 by Lammers et al. to predict the outcomes of PBC patients receiving UDCA therapy [49]. It calculates five objective variables including age at the start of UDCA therapy and levels of bilirubin, albumin, ALP, and platelet count (PLT) after 1 year of UDCA. The multicentre meta-analysis included 4119 UDCA-treated patients, at liver centers in 8 European and North American countries [49]. After 1 year of UDCA, a meta-analysis showed that only the levels of bilirubin, albumin, ALP, and PLT were independently associated with death or liver transplantation. In addition, patients with risk scores >0.30 were defined as UDCA nonresponders with significantly shorter transplant-free survival than a matched healthy individual (*p* < 0.0001) [49]. This leads to the idea that using the GLOBE score we can distinguish UDCA nonresponders, who may need second-line treatment options, from those who should continue using UDCA monotherapy. Furthermore, transplant-free survival could still be accurately calculated by the GLOBE score with laboratory values collected at 2–5 years after treatment. The limitation of this study was the exclusion of other potentially relevant PBC laboratory parameters such as prothrombin time, GGT, immunoglobulin M (IgM), or immunoglobulin G (IgG) and its relatively complex calculation [6].

### 3.4. UK Primary Biliary Cholangitis Risk Score

**Figure 1:**
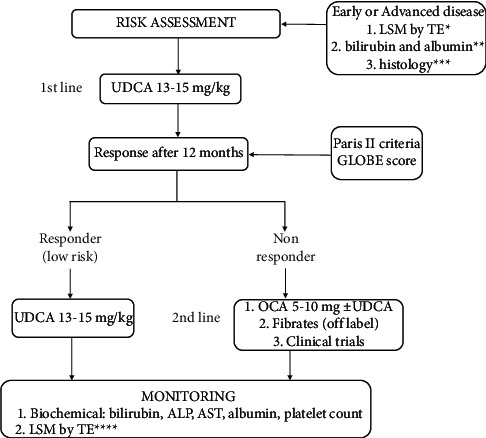
Risk assessment, treatment, and monitoring for PBC patients. ^*∗*^early from advanced disease stage based on LSM by TE (LSM <10 kPa or LSM >10 kPa). ^*∗∗*^both parameters normal vs. at least 1 parameter abnormal. ^*∗∗∗*^absent or mild fibrosis vs. bridging fibrosis or cirrhosis. ^*∗∗∗∗*^repeat TE every 2 years in early stage and every year for advanced disease.

One year after the GLOBE score was presented, Carbone et al. proposed a scoring system for a long-term prediction of end-stage liver disease (ESLD) in PBC called UK PBC risk score [50]. They analyzed data from more than 3,000 participants at liver centers in Great Britain and Northern Ireland to estimate the absolute risk of developing ESLD requiring liver transplantation at 5, 10, and 15 years from the time of diagnosis. Initial diagnosis of PBC was defined by the date of the first positive test for AMA or by the date of the diagnostic liver biopsy for seronegative patients. ESLD that requires liver transplantation was defined by 3 events: death related to liver disease (liver failure, variceal hemorrhage, or hepatocellular carcinoma (HCC)), liver transplantation for PBC, and for living patients—serum bilirubin greater than or equal to 100 mmol/L. UK PBC score includes levels of bilirubin, AST or ALT, and ALP after 12 months from diagnosis or UDCA treatment and also albumin level and platelet count at baseline as parameters of synthetic liver function and indirect signs of liver fibrosis. Since it calculates the area under the receiver operating characteristic curve (AUC) for each risk score at 5, 10, and 15 years, it is considered superior to existing prognostic models [50]. Its disadvantage could be found in the fact that they did not present a specific threshold for their risk scores. To sum up, by giving individualized, objective, and accurate information on the prognosis, this model could be used for evaluating patients who may be candidates for frequent monitoring and second-line therapies, as well as those who are at low risk of developing ESLD. The algorithm for risk assessment, treatment, and monitoring for PBC patients is presented in Figure 1.

## 4. Liver Transplantation

The LT treatment procedure includes determining an indication for LT, the process of organ allocation, and a complex surgical procedure followed by lifelong immunosuppressive treatment, whereas the main focus in the posttransplant period is aimed at the treatment of complications of the transplant procedure and immunosuppressive treatment. Since a successful outcome requires optimal patient selection and timing, the issue of which patients to list for LT and when to transplant cirrhotic patients has generated great interest and considerable controversy [51].

LT is nowadays the standard treatment procedure for all patients with end-stage acute or chronic liver failure of various etiologies, i.e., in cases where the limits of medical therapy have been reached. Evaluation for LT should be considered once a patient with end-stage liver disease or cirrhosis has experienced the first complication of portal hypertension or develops hepatocellular dysfunction resulting in a MELD score (model of end-stage liver disease) ≥15. In these patients, LT would extend life expectancy beyond that of the natural history of underlying liver disease and likely improve the quality of life (QoL). There are no uniform allocation rules or systems worldwide. Several organ exchange organizations operate in different countries and geographical areas. Most organizations have similar rules with the urgent priority group (e.g., for acute hepatic failure, early retransplantation following primary graft nonfunction, hepatic artery, or portal vein thrombosis ). In patients with chronic liver diseases, there are some differences related to organizational and allocation policies. MELD score is a good predictor of short-term pretransplant mortality risk in patients with decompensated liver cirrhosis [52]. In many Western transplant centers, the allocation of liver transplants is based on MELD score. However, not all diseases and complications are well reflected by MELD. Those patients (e.g., with HCC, refractory ascites, recurrent bleeding, encephalopathy, or intractable pruritus) should be recognized and treated differently. In most centers, priority is given to these patients by specific rules defined by multidisciplinary expert teams. Depending on the availability of the organ in specific countries and international collaboration, the waiting time on the list significantly varies.

To ensure the forehand and feasible LT, the pretransplant LT candidate workup comprises the evaluation of all potential complications of liver disease (e.g., ascites, varices, hepatic encephalopathy, hepatopulmonary syndrome, portopulmonary hypertension, hepatorenal syndrome, and hepatocellular carcinoma) and all other potential organ comorbidities. Evaluating and selecting a good recipient for LT requires the collaboration of several specialists. The final decision should be made within each expert center among a multidisciplinary team. While a potential candidate is registered on the LT list, all potentially treatable etiologies and components of hepatic decompensation should be treated and regularly evaluated.

Advances in immunosuppressive treatment, organ preservation solutions, anesthesiological and surgical procedures, and better recognition of posttransplant complications significantly improved the patient and graft survival. The average one-year survival of LT recipients is 96%, 5-year 78%, and 10-year 71% [53]. The life expectancy of transplant recipients and grafts is mostly limited by recurrent diseases such as malignant diseases and primary sclerosing cholangitis (PSC) and the occurrence of side effects associated with immunosuppression such as diabetes, chronic renal failure, hyperlipidemia, atherosclerosis, or *de novo* malignancy. For many years, there have been no new immunosuppressive drugs with lower toxicity on the horizon of transplant medicine, which further justifies efforts to better manage existing treatment options. Cholestatic liver diseases, including PBC, are considered favorable indications for LT, with 1- and 5-year patient survival rates reported between 93–94% and 82–90%, respectively. The reported rates of graft survival have been between 85 and 86% within 1 year and 81 and 82% within 5 years and are among the greatest compared with other indications [54]. Recurrence of autoimmune diseases (e.g., AIH, PBC, and PSC) varies between 10 and 50%. The exact rates of recurrence and their impact on graft function and patient survival are obscured by inconsistencies in the diagnostic approaches and criteria employed [55, 56].

## 5. Liver Transplantation Waiting List and PBC

UDCA as a recognized treatment for PBC patients has improved the natural history of the disease and its survival [57]. As a result, the number of PBC patients requiring LT has dramatically decreased over the last decades to <10% of all indications in Western countries [58].

In many other chronic liver diseases, the most common indications for LT in PBC patients are decompensated liver cirrhosis or complications secondary to portal hypertension, i.e., bleeding from gastroesophageal varices, diuretic-resistant ascites, hepatic encephalopathy, spontaneous bacterial peritonitis, and moderate hepatopulmonary syndrome when the expected survival is less than one year (MELD ≥15). Except for MELD score, another option is the Mayo risk score of 7.8 or higher. Uncontrolled and intolerable pruritus refractory to all possible medical therapies, even as an isolated indication, represents the second most common indication for LT because it provides a significant improvement in the QoL of PBC patients after LT. HCC is an exceptionally rare indication for LT in PBC patients. Although fatigue is a distinctly disabling factor, a significant proportion of patients continue to have impaired QoL after LT, and hence, it is not recognized as an indication for LT.

Before registration on the waiting list, the potential LT candidate is evaluated by a multidisciplinary team according to the standard procedure. It includes screening for complications of liver cirrhosis and portal hypertension and extensive workup for comorbidities. Even though there is no formal age limit for potential LT recipients, patients older than 65 years need a special multidisciplinary workup. In the United States, the average age of patients undergoing transplantation for PBC is in the range of 53 to 55 years. Patients are evaluated for the existence of malignant, cardiovascular, renal, pulmonary, oropharyngeal, urological, gynecological, and psychiatric diseases. Finally, they are evaluated for their nutritional and overall functional status and the presence of osteoporosis (Table 2). PBC patients often have associated autoimmune and metabolic diseases, especially hyperlipidemia and osteoporosis, thyroid disease, keratoconjunctivitis sicca, and xerostomia. Those persist or even may worsen after LT and should be properly treated. Hyperlipidemia in PBC patients is common and yet it has not been shown to carry additional cardiovascular risk in the absence of other risk factors for cardiovascular disease (CVD). Part of the confusion appears from the various effect of PBC on lipid metabolism. In early disease, patients often have elevated levels of LDL (which increases cardiovascular risk) and HDL levels and also levels of adiponectin and lipoprotein X (Lp-X), a circulating lipid particle with a density similar to LDL, which has a cardioprotective effect [60]. Also, PBC therapy impacts lipid metabolism in a way that UDCA increases cholesterol absorption and fibrates are modulating bile acid and cholesterol transportation [60]. Lipid-lowering therapy should be individualized based on CVD risk assessment and comorbidities and currently published guidelines are not offering strong recommendations regarding monitoring or treatment. Statins are the first choice for therapy, and since data on risk stratification within PBC are not available and most studies have only examined moderate-intensity statins (atorvastatin 10–20 mg daily or simvastatin 20–40 mg daily), it is safe at these doses and up titrate as clinically indicated [61, 62]. Although fibrates are a promising therapy for PBC, in the context of hyperlipidemia treatment they have no advantage in lowering overall cardiovascular morbidity and mortality over statins. A meta-analysis of fibrates did show a 10% relative risk reduction (95% CI, 0 to 18) in major cardiac events but did not improve cardiovascular mortality [63]. Metabolic bone disease (osteopenia, osteoporosis) is a common complication of PBC, which increases morbidity and mortality [64, 65]. Therapeutic options are limited and mostly derived from osteoporosis in postmenopausal women. PBC-related osteoporosis is driven primarily by decreased bone formation compared with postmenopausal osteoporosis, which is secondary mostly to increased bone resorption [66]. Patients after liver transplantation are prone to osteopenia and osteoporosis, with an expected bone loss of 8% to 18% in the first 3–6 months after liver transplantation [67, 68] and 20% to 40% incidence of fractures in the first year posttransplant [67, 69]. Prevention and treatment of osteoporosis before and after transplantation are imperative in the overall management of PBC. It is suggested that all patients undergo bone mineral density assessment (dual-energy X-ray absorptiometry (DEXA)) at the time of diagnosis and continue with surveillance between 1 and 5 years later depending on the outcome and general osteoporosis risk [1]. Preventive measures include optimal lifestyle and nutritional support. Supplementation of vitamin D and calcium is recommended by EASL guidelines in all PBC patients without a history of renal stones [1]. Many treatment strategies for osteoporosis in PBC are copied from therapeutic options in postmenopausal osteoporosis. Several trials have demonstrated that bisphosphonates, especially weekly alendronate, and monthly ibandronate, are effective in increasing bone mass in patients with PBC [70]. Additional studies investigating PBC-specific therapies with a focus on improving bone formation are necessary to improve patients' outcome.

Patients with cirrhosis are especially prone to various clinically evident and latent infections that could result in the development of multiple organ failures and death before and after LT. Screening for bacterial, fungal, and viral acute or chronic infections is mandatory before LT. The presence of an active uncontrolled infection contraindicates the procedure. The infectious screening should be performed at all time points in the process of LT: in all LT candidates, in patients eligible for LT at the time of listing, and in patients with risk factors according to their clinical history, comorbidities, and exposure to endemic diseases (Table 2). Regarding vaccination, it is important to make sure that LT candidates are immunized against HAV and HBV, varicella, Pneumococcus, influenza, and tetanus, and concerning the current epidemiological situation, COVID-19.

Pretransplant assessment is not uniform to all transplant teams, and the optimal approach is constantly evaluated and changing in each transplant center. Absolute and relative contraindications to LT are also changing over time and may vary among liver transplant centers, depending on their local expertise.

Patients on the waiting list should be regularly evaluated and properly treated for the consequences of portal hypertension and liver decompensation.

## 6. Recurrent Primary Biliary Cholangitis

In approximately 21% to 37% of patients who have undergone liver transplantation as the only definitive treatment for PBC, recurrence of the disease was reported after 10 years. [6]. Initial studies showed a lower incidence of disease recurrence, but with long-term follow-up, rPBC was reported by most world centers with growing numbers [71]. Data from multiple studies considering median time to graft loss as a consequence of disease recurrence showed no difference in survival of patients with recurrence of the disease in contrast to those without it. Nevertheless, with time, there is a possibility of this becoming a greater challenge in the long-term treatment of patients [6, 71].

### 6.1. Diagnosis of Recurrent PBC

Diagnosis of rPBC comes with a set of challenges in comparison with the diagnosis of PBC, which is mainly because clinical and serological findings are not as useful as in diagnosing *de novo* disease so clinicians depend on histopathological findings, which are received with performing invasive procedures and consequently not routinely done. According to the American Association for the Study of Liver Diseases, de novo PBC is diagnosed in case of long-term elevated ALP serum levels in combination with one of the other criteria: either positive AMA antibodies, positive PBC-specific ANA, or histopathological findings affirmative of PBC [3, 6, 71].

#### 6.1.1. Clinical Features

Characteristic symptoms of PBC in the native liver are not necessarily present in the recurrent forms of the disease. Moreover, studies show that clinical manifestations of the disease, such as chronic fatigue and pruritus, surface in only 12% of patients with a confirmed diagnosis. Furthermore, concomitant autoimmune diseases (thyroid disease, keratoconjunctivitis sicca, and xerostomia) may persist and/or resolve after transplantation or even from *de novo,* but none of these is a predictive factor for disease recurrence [6, 71, 72].

#### 6.1.2. Serology Features

Persistent elevation of cholestatic parameters such as ALP combined with either positive AMA antibodies or positive PBC-specific ANA is enough for a serologic diagnosis of PBC, however in rPBC that is not the case. Approximately 50% of patients with normal liver biochemistry may have characteristic histology finding on protocol biopsy. Additionally, in cases where the diagnosis was made on histologic findings in the allograft, it was not mirrored by the cholestatic profile of liver enzymes. Another contributing factor to ALP non-specificity is a large number of conditions with ALP elevation after transplantation, including acute and chronic graft rejection, viral infections, graft-versus-host disease, or obstructive cholestasis. AMA nor PBC-specific ANA also cannot be used in the diagnosis of recurrent types of the disease since their role in diagnosing rPBC is limited [6, 27, 28, 73]. After transplantation, there is usually a transient fall in serum levels of both AMA and PBC-specific ANA, but in the long term, their levels in the majority of patients stay elevated [74–76].

#### 6.1.3. Histology Features

Liver biopsy and characteristic histological findings are the only valid parameters for the diagnosis of recurrent PBC. Not all centers require protocol allograft biopsies in long-term follow-up of transplanted patients with PBC, which could falsely lead to lower reported rates of rPBC. A good marker for the necessity of liver biopsy could be the elevation of IgM levels, considering that it has been shown that IgM levels are more likely to be elevated in patients with recurrence of PBC after transplantation than in those without it [6, 71]. Florid duct lesions or destructive lymphocytic cholangitis presence is defined as a histologic hallmark of disease recurrence. To be exact, there are four specific portal tract lesions: damage to the bile ducts, lymphoid aggregate formations, and the presence of mononuclear inflammatory infiltrate or epithelioid granulomas. If two of four of these characteristics are present in the liver biopsy, a diagnosis of rPBC is highly probable. If all of them are recognized, then the diagnosis is definitive [6, 77]. Even with histopathological characteristics of rePBC, other causes of graft failure must be excluded, such as acute and chronic rejection of an allograft, viral infections (CMV, HCV), and graft-versus-host disease. The Birmingham study published in liver transplantation showed that in 13 of 83 biopsy specimens taken from patients transplanted for PBC, a recurrent form of the disease was diagnosed. However, in 12 of them, a histologic stage of 1-2 was established and only one patient developed cirrhosis in the liver allograft. There is the utmost importance of follow-up biopsies in patients with the histological finding of stages 1 and 2 to determine disease progression and timely diagnosis [71]. Sylvestre and colleagues in the study done at the Mayo Clinic have confirmed that one-half to one-third of patients with a definitive histological diagnosis of rPBC had normal ALP levels at the time of biopsy [77].

In summary, diagnostic criteria for rPBC include anamnestic data of liver transplantation for PBC, positive serum levels for AMA or PBC-specific ANA with the existence of mononuclear inflammatory infiltrate, lymphoid aggregates, epithelioid granulomas, and bile duct destruction with pathohistological findings of liver biopsy, all of which is preceded with the exclusion of other causes for graft failure [6, 71, 73, 76].

## 7. Risk Factors for Recurrence

Over the years, a large number of risk factors for the recurrence of PBC have been analyzed and many of them remain controversial. Patients undergoing liver transplantation for PBC are usually in their 60 s or 70 s. Certain studies showed a positive correlation between younger recipients' age and a higher rate of recurrence, while a study published by Silveira and colleagues saw a greater risk of recurrence in patients who were older at the time of LT [72]. The role of HLA mismatch as a risk factor for PBC recurrence also remains controversial, but Sanchez and colleagues concluded that certain patterns of alleles are found more often in patients with rPBC, such as A1, B57, B58, DR44, DR57, and DR58 in donors and B48 in recipients [78]. According to one Japanese study [79], a small number of mismatches in HLA-A, HLA-B, and HLA-DR were associated with a higher risk of PBC recurrence, and a high number of those are connected to increased mortality 6 months after transplantation. Factors of the donor liver, such as age and warm and cold ischemic time, were also analyzed. In a study published by Silveira and colleagues, a donor older than 65 years was described as a risk factor in the case of tacrolimus immunosuppression. Cold ischemic time was recognized as a risk factor, while warm ischemic time was not described as statistically significant [72]. The use of different calcineurin inhibitors in immunosuppression therapy after transplantation was also evaluated. In a few studies, with the use of tacrolimus, a shorter time from transplantation to recurrence has been described in comparison with cyclosporine [72, 78, 80]. Corticosteroid therapy also seems to have a role in rPBC. Several studies showed that immunosuppressive therapy without corticosteroids may increase the incidence of recurrence [71, 72].

Until recently, the results of several studies showed that rPBC has a limited overall impact on graft or recipient survival and all studies had an evident limitation in the short follow-up period [56, 81, 82]. A retrospective, multicentre study published by Montano et al. [82] was the first to demonstrate that recurrence of PBC was significantly associated with graft loss (HR, 2.01; 95% CI, 1.16–3.51) and death of recipient (HR, 1.72; 95% CI, 1.11–2.65). The same study also showed that the age at diagnosis <50 years, age at liver transplantation <60 years, use of tacrolimus, and biochemical markers of severe cholestasis (bilirubin >100 mmol or alkaline phosphatase >3-fold the upper limit of normal) at 6 months after liver transplantation were associated with a higher risk of PBC recurrence, while the use of cyclosporine reduced risk of rPBC. The only available treatment option for rPBC is UDCA, and there is numerous observational evidence that re-induction of UDCA leads to biochemical improvement [50]. Some centers started using UDCA preemptively to reduce the incidence of rPBC and biliary complications after LT. In 2015, retrospective multicentre analysis showed that preventive administration of UDCA was associated with a significant reduction (21% vs. 62%) in the risk of PBC recurrence over the 10-year follow-up [83]. The effect of preventive exposure to UDCA on the incidence and long-term impact of rPBC after LT was investigated in the longitudinal retrospective study that included the largest cohort of transplanted patients with PBC to date [84]. The study showed that preventive exposure to UDCA (10–15 mg/kg per day) was associated with reduced risk of rPBC (adjusted HR (aHR) 0.41; 95% CI, 0.28–0.61; *p* < 0.0001), graft loss (aHR, 0.33; 95% CI, 0.13–0.82; *p* < 0.05), liver-related death (aHR, 0.46; 95% CI, 0.22–0.98; *p* < 0.05), and all-cause death (aHR, 0.69; 95% CI, 0.49–0.96; *p* < 0.05). The beneficial effect of cyclosporin over tacrolimus was also confirmed in this study. Moreover, the combination of preventive UDCA and cyclosporine was associated with survival gains of 2.26 years (95% CI, 1.28–3.25) and 3.51 years (95% CI, 2.19–4.82), respectively, over 20 years. The exact mechanism of action involved in the preventive effect of UDCA on rPBC is unclear, but it is assumed to be related to the well-known immunomodulatory and anti-inflammatory properties such as inhibiting prostaglandin E2 (PGE2), thus blocking the propagation of autoimmune liver injury and decreasing the hepatocellular expression of MHC class I and the biliary expression of MHC class II, thus interfering with the autoimmune basic mechanisms [44, 84]. Recurrence of primary biliary cholangitis is relatively common; luckily, many patients are diagnosed with a histologic stage of 1-2 and very rarely there is a need for retransplantation. In a large study including 486 patients who underwent LT for PBC, only 2 of them again reached end-stage liver disease caused due to rPBC and were retransplanted [85], but Corpechot and colleagues have shown that in a prolonged follow-up period, rPBC has a significant impact on graft and recipient survival [84]. Additional studies (preferable randomized clinical trials) are needed to confirm the beneficial effects of UDCA and immunosuppressive regime on rPBC and to explore the usefulness and effects of current second-line therapies for PBC (OCA and fibrates) in the context of rPBC.

## 8. Conclusion

Diagnosis of PBC can be made using biochemical and serologic findings, and easier diagnostic requirements result in increasing prevalence and incidence of the disease. On the other hand, available treatment options, especially UDCA, changed the clinical course of the disease and prolonged LT-free survival, and there is no increased incidence of patients with PBC added to the waitlist for LT. Several risk scores are proposed for better evaluation of patients before and during treatment to stratify patients at increased risk of disease progression and ESLD development. GLOBE score and UK PBC risk score are widely used for the evaluation of UDCA treatment with the greatest advantage of not needing a liver biopsy to evaluate the treatment's effect, only noninvasive objective data. For UDCA-refractory or UDCA-intolerant PBC, OCA has been approved as a second-line treatment and there are ongoing trials for several new treatment options. LT is the only treatment option in the case of ESLD. Up to 40% of patients experience recurrence of the disease more than 10 years after LT. rPBC is a histological diagnosis, and liver biopsy is required. Several studies highlighted potential risk factors for rPBC such as the role of HLA mismatching, use of corticosteroids after LT, or type of calcineurin inhibitors but with no strong conclusions. Until recently, it was considered that rPBC has a limited overall impact on recipient and graft survival mostly due to the short follow-up period. Now, we have several studies with longer follow-up periods that demonstrated that rPBC is significantly associated with graft loss and death of the recipient. With this in mind, there is a need to find an effective therapy for rPBC and if possible, to prevent disease recurrence. The use of UDCA after rPBC is associated with biochemical and clinical improvement in the majority of patients, and recently published studies even show a beneficial effect of UDCA use after transplantation for the prevention of rPBC development. Further studies are needed to rule on the preventive effect of UDCA on rPBC and to make conclusions on universal prophylactic therapy after LT. LT is a definitive treatment option for ESLD, but the question arises as to what can be done to prevent the progression of the disease. Although the use of UDCA has significantly altered the natural course of PBC, about 40% of patients have an inadequate clinical response and are at high risk of disease progression. Furthermore, currently approved therapies for PBC do not affect frequent clinical symptoms such as pruritus and fatigue, and additional therapy for symptom control is often not enough. Moreover, intractable pruritus with all available symptom control therapies is an indication for liver transplantation. There are multiple ongoing trials to address the lack of treatment options for PBC, and fibrates appear to be the most promising new therapy in achieving PBC treatment endpoints.

## Figures and Tables

**Table 1:** Prognostic models for PBC.

Prognostic models	Year	Settings	Sample size (*n*)
Pre-UDCA era			
Yale model [40]	1983	USA	280
European model [41]	1985	Denmark	248
Mayo score [42]	1989	USA	418
*UDCA era*			

Barcelona criteria [43]	2006	Spain	192
Paris I criteria [44]	2008	France	292
Rotterdam criteria [45]	2009	Netherlands	375
Toronto criteria [37]	2010	Canada	69
Paris II criteria [46]	2011	Spain	165
APRI score [47]	2014	Britain	1015
ALBI score [48]	2015	China	61
GLOBE score [49]	2015	Netherlands	4119
UK PBC risk score [50]	2016	UK	1916

**Table 2:** Comorbidity assessment in liver transplant candidates.

Comorbidity	Procedure	Associated risk
Cardiovascular	ECG, heart ultrasound with Doppler ergometry or pharmacological stress test (>50 years or with multiple cardiovascular risk factors for coronary heart disease), coronary angiography (with positive ergometry test or pharmacological stress test)	In the case of adequately treated coronary heart disease, the risk is equal to the rest of the population, for recipients aged >70 years increased cardiovascular risk
Respiratory	Chest X-ray, spirometry, diffusion capacity for CO, the definition of hepatopulmonary syndrome (HPS; calculation of alveolar/arterial oxygen gradient or contrast echocardiography) and portopulmonary hypertension (PPHTN; mean pulmonary artery pressure—MPAP >30 mmHg, right-sided cardiac catheterization is obligatory)	For HPS and pO2 <50 mmHg without response to 100% oxygen therapy—possible irreversible respiratory failure not corrected with LT, for PPHTN and MPAP ≥35 mmHg not responding to pulmonary vasodilator therapy—high perioperative mortality MPAP 35–50 mmHg—50% risk of mortality after LT [59] MAP >50 mmHg absolute contraindication for LT—100% risk of posttransplantation mortality [59]
Renal	Abdominal and kidney ultrasound, spot urine test, K/Na/protein/creatinine in daily urine, eGFR (MDRD6)	Sevenfold increased perioperative risk recipients with GFR <30 mL/min or hepatorenal syndrome and dialysis >8–12 weeks or >30% glomerulosclerosis or fibrosis on kidney biopsy—simultaneous liver and kidney transplantation indicated
Nutritive status	Body mass index (BMI), prealbumin, psoas thickness (MSCT)	Recipients with a BMI <18.5 or >40 have elevated mortality
Osteoporosis	Densitometry	Osteoporotic fracture (fractures of the hip, vertebrae, and distal forearm are the most common)
Infections	The first level of screening consists of screening for human immunodeficiency virus (HIV) 1 and 2 antibodies, HBV serology, HCV antibodies, HAV antibodies, cytomegalovirus (CMV), and completing a chest X-ray [51] The second level of screening consists of screening for *Mycobacterium tuberculosis* (history + PPD-Mantoux + IFN gamma release assays), Epstein–Barr virus (EBV), human herpes virus 8 (HHV-8), varicella-zoster virus (VZV), herpes simplex virus 1 (HSV-1), herpes simplex virus 2 (HSV-2), urine culture, parasitological examination and stool culture (*Strongyloides stercoralis* serology, *Toxoplasma gondii* IgG, *Treponema pallidum* serology), Immunoenzymatic Assay with Venereal Disease Research Laboratory (VDRL), *Staphylococcus aureus* nasal/axillary swab, and dentist review recipients should receive the vaccine for HAV, HBV, chickenpox, pneumococcus, influenza, tetanus, COVID-19	Uncontrolled sepsis, bacterial, viral, and invasive fungal infections (aspergillosis) are a contraindication for the LT procedure
